# Diagnosis of Cryptococcosis and Prevention of Cryptococcal Meningitis Using a Novel Point-of-Care Lateral Flow Assay

**DOI:** 10.1155/2013/640216

**Published:** 2013-11-12

**Authors:** Ashar Dhana

**Affiliations:** Department of Infectious Diseases, Helen Joseph Academic Hospital, 1 Perth Road, Westdene, Johannesburg 2092, South Africa

## Abstract

Despite access to antiretroviral therapy, mortality from cryptococcal meningitis (CM) is high among persons with advanced HIV infection in sub-Saharan Africa. Cryptococcal antigen (CrAg) is present several weeks to months before the onset of symptoms of meningitis and can be screened to prevent life threatening meningitis. Recently, the World Health Organisation recommended that a new rapid CrAg lateral flow ‘‘dipstick” assay (LFA) is to be used to screen HIV-infected persons with CD4 counts of less than 100 cells/*µ*L. In this paper, we describe two cases of cryptococcosis with differing outcomes. In the first case, the new CrAg LFA was used as part of a screen and preemptive treatment strategy to prevent CM. In the second case, our patient had no access to the CrAg LFA and subsequently developed life threatening meningitis. To the best of our knowledge, this is the first case report of cryptococcosis diagnosed using this novel assay.

## 1. Introduction

Cryptococcosis is an infection caused by fungi that belong to the genus *Cryptococcus*. Two species in particular—*C. neoformans *and* C. gattii*—cause nearly all cryptococcal infections in humans [1]. *C. neoformans* causes most cryptococcal infections in immunocompromised patients, while *C. gattii* causes disease in immunocompetent and, to a lesser extent, immunocompromised persons. Both species can infect any organ in the body, but most often they infect the lungs or invade the central nervous system, causing life threatening meningitis. Compared to *C. neoformans*, however, *C. gattii *is more commonly associated with isolated pulmonary infection, neurologic sequelae, and decreased in vitro susceptibility to common antifungal drugs [2, 3]. 

Cryptococcal meningitis (CM) is common in patients with advanced HIV infection, affecting an estimated 957,900 people per year [4]. Over 70% of these cases occur in sub-Saharan Africa, which bears a disproportionate burden of disease; CM is now the most common cause of meningitis in this setting [5]. Even in parts of the world with access to antiretroviral therapy (ART), mortality from CM is high with only 40–60% of patients being alive at 6 months [4, 6]. 

Current diagnostic tests for cryptococcosis include antigen tests and culture. Culture is the gold standard but is too insensitive [7]. Antigen tests are more commonly used to detect cryptococcal antigen (CrAg) by either latex agglutination (LA) or enzyme immunoassay (EA). These tests are sensitive and specific but require technical expertise, a central reference laboratory, and special storage [8]. Recently, the CrAg lateral flow assay (LFA)—a new rapid “dipstick test”—was developed to overcome problems with the diagnosis of CM in resource limited settings. Besides facilitating the early diagnosis of CM, the CrAg LFA shows great potential to prevent CM through targeted screening of HIV-infected individuals with advanced immunodeficiency [9]. We report two cases of cryptococcosis with contrasting outcomes and discuss the role of the new CrAg LFA in the diagnosis and prevention of CM. 

## 2. Case Presentation 1

A 38-year-old male was seen at his local clinic with a 6-month history of loss of weight (approximately 10 kg) and progressive loss of appetite. A rapid HIV test was performed and was positive. Additional blood tests confirmed a CD4 cell count of 2 cells/*μ*L and a positive CrAg LFA. Targeted screening of patients with CD4 cell counts of less than 100 cells/*μ*L for cryptococcal antigenemia was recently introduced into selected laboratories in South Africa as part of the National Strategic Plan on HIV, STIs, and TB. After a positive serum CrAg LFA test, the patient was referred to our hospital for a lumbar puncture to exclude CM. He reported no headache, neck stiffness, fever, visual impairment, or night sweats. Although he had a productive cough approximately 3 weeks ago, it had resolved with antibiotics prescribed by his local clinic. On examination, temporal wasting and generalized lymphadenopathy were present. Neurological examination revealed no neck stiffness, focal neurologic deficit, papilledema, or cranial nerve involvement. No cutaneous lesions or pulmonary signs were present. The remainder of the systemic examination was normal.

Laboratory blood tests showed a normocytic anaemia with haemoglobin of 11.8 g/dL. The cerebrospinal fluid (CSF) on admission was clear in appearance, and further analysis revealed lymphocytes of 1 cells/*μ*L, neutrophils of 0 cells/*μ*L, protein of 0.48 g/dL (normal: 0.15–0.45 g/dL), and glucose of 3.2 mmol/L (normal: 2.8–4.4 mmol/L). CSF Gram's stain, CrAg latex antigen (LA), and India ink were all negative. Xpert MTB/Rif assay of early morning sputum samples was negative for tuberculosis. A chest radiograph showed no abnormalities.

A diagnosis of disseminated cryptococcosis was made. Antifungal therapy was started with high dose fluconazole of 800 mg daily according to the South African screen-and-treat algorithm for ART-naïve patients with a positive CrAg and negative lumbar puncture. Cotrimoxazole was also given as prophylaxis to prevent pneumonia from Pneumocystis *jiroveci*. The patient was discharged from hospital a day after his admission. At the 2-week follow-up visit, the fluconazole dose was reduced to 400 mg daily and ART—with tenofovir, emtricitibine, and efavirenz—was initiated. At the 2-month visit, the fluconazole dose was further reduced to a maintenance dose of 200 mg daily. So far he is doing well and reports no symptoms of meningitis.

## 3. Case Presentation 2

A 37-year-old female presented to the emergency department with a 1-week history of progressive neck stiffness, severe headache, fever, and loss of weight. She reported no other neurological symptoms. She had no medical history of TB but was diagnosed with HIV two years ago. At a visit to her local clinic about 3 weeks before this admission, laboratory tests showed a CD4 cell count of 76 cells/*μ*L. She was subsequently started on antiretroviral therapy—with tenofovir, emtricitabine, and efavirenz. Targeted screening for cryptococcal antigenemia was not yet offered by her clinic. The physical examination revealed tachycardia, pyrexia, conjunctival pallor, and generalized lymphadenopathy. Neck stiffness was present, but no confusion, focal neurologic deficit, papilledema, or cranial nerve involvement was evident on neurological examination. No pulmonary or dermatological changes were noted. The remainder of the systemic examination was otherwise normal.

The initial CSF opening pressure was raised (28 cm H_2_0). CSF was clear in appearance, and further analysis revealed lymphocytes of 34 cells/*μ*L, neutrophils of 5 cells/*μ*L, protein of 1.83 g/dL (normal: 0.15–0.45 g/dL), and glucose of 1.9 mmol/L (normal: 2.8–4.4 mmol/L). Gram's stain of the CSF was negative. The cryptococcal latex antigen test (CLAT) and India ink were, however, positive. CSF CrAg titres are not routinely performed in South Africa, as antifungal treatment is standardised and fungal burden at the time of diagnosis is invariably high. Other laboratory tests showed a positive peripheral blood CrAg LFA and a normocytic anaemia (haemoglobin: 9.2 g/L). Chest radiography was normal.

A diagnosis was made of cryptococcal meningitis with raised intracranial pressure and unmasking HIV-associated IRIS. ART was continued in the ward, and antifungal therapy was started with amphotericin B (1 mg/kg/day IV) and high dose fluconazole of 800 mg daily. Fluconazole is used in South Africa, as flucytosine is presently unavailable. Intravenous antimicrobial therapy with ceftriaxone was also started. To avoid nephrotoxicity and electrolyte abnormalities, she was prehydrated and given potassium and magnesium supplements. No nephrotoxicity or worsening of anaemia was noted during admission. Therapeutic lumbar puncture was performed three times a week to relieve symptoms of raised intracranial pressure, and paracetamol was given for further analgesia. Corticosteroids were also added to the regimen. By hospital day 14, her symptoms had improved greatly. Intravenous amphotericin was stopped and the dose of fluconazole was changed to 400 mg daily. She was discharged from hospital 3 weeks after admission. At her 2-month follow-up visit, she was maintained on fluconazole of 200 mg daily as secondary prophylaxis. She is currently well and reports no recurrence of her symptoms. 

## 4. Discussion

These cases demonstrate the utility of the new CrAg LFA assay in the diagnosis of cryptococcosis and prevention of CM. 

A large proportion of CM is preventable for several reasons. First, CrAg is present in peripheral blood for an average of 22 days before the onset of CM, and in 10% of cases it may be present for more than 100 days before the onset of meningitis [10]. As seen with our first patient, this asymptomatic phase is important for identifying those at risk of developing clinical disease at a later stage. Second, in HIV-infected individuals with CD4 counts of less than 100 cells/*μ*L, up to 12% have been shown to be CrAg positive [8]. One retrospective study showed that almost 30% of these CrAg positive patients will go on to develop CM [11]. Finally, in one study, over 70% of patients who were diagnosed with CM already knew their HIV status. Moreover, many of these patients were recently tested for HIV (within the last 4 months), but the diagnosis of cryptococcosis was missed [12]. As a result, it has become clear that screening for CrAg in advanced immunodeficiency plays an important role in any ART programme.

**Figure 1:**
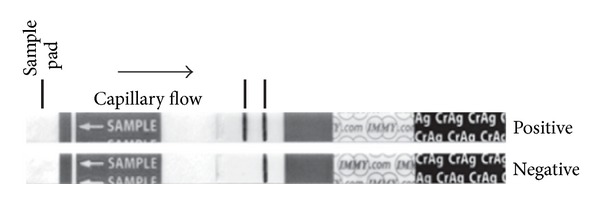
The cryptococcal antigen lateral-flow assay is read in the same way as that of a urine pregnancy dipstick test. The presence of two visible bands indicates a positive result.

Recently, the newly developed CrAg LFA (Figure 1) has shown promise as a diagnostic and screening tool for cryptococcosis, meeting most of the WHO criteria for an ideal point-of-care test [13]. The assay provides a result in less than 10 minutes and requires no special storage or prior laboratory expertise. Of four prospective and retrospective studies, the CrAg LFA showed excellent diagnostic accuracy when compared to culture and CrAg LA or EA [9, 14–16]. On CSF and peripheral blood samples, sensitivity ranged from 96% to 100% and specificity was 92% to 100%. In these studies, agreement and correlation between the CrAg LFA and other diagnostic assays were also shown to be very high. The assay also shows acceptable sensitivity (70–98%) with urine samples, which are easier to collect and pose minimal biohazard risk [9, 14, 15]. Furthermore, at a cost of $2.50, the CrAg LFA is less than half the cost of the more common assays [15]. In one prospective study in Uganda, screening with the CrAg LFA was found to be more cost-effective than a prophylactic course of antifungal treatment in HIV-infected patients with advanced immunodeficiency [17]. 

Early diagnosis of cryptococcosis—either subclinical or clinical—is a crucial aspect of infectious disease control, especially in resource limited settings. The new CrAg LFA shows potential as a point-of-care test and may substantially reduce the global burden of cryptococcal meningitis. Given the high morbidity and mortality of CM, further studies are needed on how to best implement this novel test into existing ART programmes and diagnostic algorithms.
